# SPRi-Based Biosensing Platforms for Detection of Specific DNA Sequences Using Thiolate and Dithiocarbamate Assemblies

**DOI:** 10.3389/fchem.2018.00173

**Published:** 2018-05-22

**Authors:** Marcin Drozd, Mariusz D. Pietrzak, Elżbieta Malinowska

**Affiliations:** ^1^Faculty of Chemistry, The Chair of Medical Biotechnology, Warsaw University of Technology, Warsaw, Poland; ^2^Centre for Advanced Materials and Technologies, Warsaw, Poland

**Keywords:** DNA biosensors, surface plasmon resonance imaging, DNA immobilization, self-assembled monolayers, dithiocarbamate, gold functionalization

## Abstract

The framework of presented study covers the development and examination of the analytical performance of surface plasmon resonance-based (SPR) DNA biosensors dedicated for a detection of model target oligonucleotide sequence. For this aim, various strategies of immobilization of DNA probes on gold transducers were tested. Besides the typical approaches: chemisorption of thiolated ssDNA (DNA-thiol) and physisorption of non-functionalized oligonucleotides, relatively new method based on chemisorption of dithiocarbamate-functionalized ssDNA (DNA-DTC) was applied for the first time for preparation of DNA-based SPR biosensor. The special emphasis was put on the correlation between the method of DNA immobilization and the composition of obtained receptor layer. The carried out studies focused on the examination of the capability of developed receptors layers to interact with both target DNA and DNA-functionalized AuNPs. It was found, that the detection limit of target DNA sequence (27 nb length) depends on the strategy of probe immobilization and backfilling method, and in the best case it amounted to 0.66 nM. Moreover, the application of ssDNA-functionalized gold nanoparticles (AuNPs) as plasmonic labels for secondary enhancement of SPR response is presented. The influence of spatial organization and surface density of a receptor layer on the ability to interact with DNA-functionalized AuNPs is discussed. Due to the best compatibility of receptors immobilized via DTC chemisorption: 1.47 ± 0.4 · 10^12^ molecules · cm^−2^ (with the calculated area occupied by single nanoparticle label of ~132.7 nm^2^), DNA chemisorption based on DTCs is pointed as especially promising for DNA biosensors utilizing indirect detection in competitive assays.

## Introduction

Surface of gold transducers due to numerous attractive features, such as the repeatability of their preparation, excellent electrochemical properties, and unsurpassed chemical stability have gained unflagging interest as platforms for immobilization of protein- and oligonucleotide-based receptors (Sendroiu et al., 2011; Choi et al., 2014). Rapid and high-throughput detection of DNA is in high demand in many areas such as medical diagnostics (e.g. single nucleotide polymorphism, disease markers, and pathogens detection), DNA sequencing, and environmental monitoring (Mannelli et al., 2003; Fiche et al., 2008; Kong et al., 2014; Pursey et al., 2017). Facility of preparation of ultra-thin Au-layers on glass substrates has opened up the possibility for a development of relatively simple and routine methodology of optical transducers fabrication. Capability of contactless, spatially-resolved monitoring of surface plasmon resonance (SPR) (its angle shift) on a very small area using imaging mode enabled simultaneous real-time detection and determination of various analytes with a use of different receptors spotted on a surface of transducers. Such multiplexed screening approach underlies the SPRi-based DNA biosensors and arrays (Nelson et al., 2001; Chen et al., 2009).

To take full advantage of multiplexed detection of specific DNA sequences, efforts for a development of methods of robust and regioselective tethering of oligonucleotide probes to gold have been made. Such applications require both the high durability of bioreceptor-Au transducer coupling and possibly high surface density of DNA probes (Keighley et al., 2008). The thoughtful choice of linkage chemistry is particularly important due to the need to ensure the compatibility with the methods of multispotting, typically used for fabrication of sensing platforms for high-throughput screening (Brockmann et al., 1999; Leonov and Wei, 2011; Simon et al., 2015). Among methods of gold biofunctionalization, these based on monolayers of compounds capable of chemisorption via terminal, sulfur-containing groups, mainly thiol derivatives, and more rarely other such as disulfides, sulfides, thioesters, isothiocyanates, or dithiocarbamates (DTCs) (Ulman, 1996; Mandler and Kraus-Ophir, 2011). Despite the common use of thiolate chemisorption, there is a number of reported disadvantages, limiting the use of such strategy for a construction of functional receptor layers for biosensing (Schoenfisch and Pemberton, 1998; Raigoza et al., 2012). Park et al. (2010) reported the lability of monodentate linkage of alkanethiols with gold, which negatively affects the resolution of patterns obtained via thiol self-assembly on Au substrates. Another significant difficulty in the case of direct immobilization of biologically originated compounds is a shortage (or even a total absence) of exposed thiolate moieties, which enforces the necessity of chemical modification of such receptors prior to the immobilization. Because of the listed disadvantages and due to the susceptibility of Au-thiolate bond to photochemical oxidation as well as relatively low resistance to desorption when in contact with surface-active agents (such as biogenic thiols), it seems reasonable to develop alternative, preferably one-step method to tether DNA-probes to the gold surface.

A DTC chemisorption on gold was reported as promising alternative to thiol self-assembly for the first time by Zhao et al. (2005). Since that time, bidentate anchoring of bifunctional DTC derivatives has gained interest as the elegant way of both fabrication of sensing platforms and functionalization of nanomaterials (Sharma et al., 2008; Adak et al., 2010; Wang et al., 2012). It was reported, that low-molecular weight DTCs are capable to form bonds with gold of the strength high enough to competitively desorb short thiols. It offers new possibilities to design orthogonal assemblies for further deposition of biomolecules, nanoparticles and dyes (Park et al., 2010; Leonov and Wei, 2011). The important advantage of DTC chemistry, in terms of direct immobilization of bioreceptors, is the facility of DTC synthesis in a reaction between primary or secondary amines with carbon disulfide according to S_N_2 substitution mechanism (Morf et al., 2006). The common presence of receptors bearing amine moieties (proteins, oligopeptides) and commercial availability of oligonucleotides modified with terminal aminolink significantly extends the pool of available bioreceptors compatible with this immobilization method in comparison to direct immobilization of thiols.

Recently, direct immobilization of vital components of biosensors via DTC linkage was successfully demonstrated for various types of proteins: protein A (Paiva et al., 2017) and enzymes such as laccase (Almeida et al., 2017) and glucose oxidase (Almeida et al., 2012). In each case naturally occurring amine groups were converted to respective DTCs prior to direct attachment of such biomolecules to gold. Despite the increasing importance of DTC-Au chemistry for fabrication of biosensors, only two works focusing on assembly of short DNA oligonucleotides terminated with ω-aminoalkyl moiety can be found in the literature. First of them refers to electrochemical (Wang et al., 2012), while the second one to chemiluminescent (Lou et al., 2015) DNA biosensor. Importantly, although the use of bidentate DTCs is indicated as the important advantage, no attempt was made to directly compare physicochemical and analytical performance of DTC-DNA assemblies to analogous monolayers based on thiolated DNA assemblies in the reported studies.

In the framework of presented studies we examined three different strategies of regioselective attachment of model ssDNA probes for fabrication of SPRi biosensors, including two chemical approaches: DTC and thiolate chemisorption, and physisorption of unmodified oligonucleotides. The special emphasis was put on the critical comparison of the analytical parameters of obtained biosensing platforms when detecting the target sequence. SPRi studies were performed both in the label-free format as well as applying a signal enhancement strategy with a use of DNA-functionalized AuNPs as plasmonic labels. The main aim of this work was to develop general methodologies, which are useful for preparation of receptor layers utilizing hybridization as the recognition mechanism. Biosensors constructed on their base can find applications in analytics for detection of short oligonucleotides, such as specific genomic sequences, microRNA, or PCR products.

## Materials and methods

### Chemicals and oligonucleotides

6-mercapto-1-hexanol (MCH), carbon disulfide, hexaamineruthenium(III) chloride (RuHex), and tris(hydroxymethyl)aminomethane (Tris) were obtained from Sigma-Aldrich (Poland) and used as received. Perhydrol, sulfuric acid (96%), ammonia (25%), and ethanol (96%) were purchased in Avantor S.A. (Poland). All chemicals used in this work were at least analytical grade. Milli-Q water (DI) (resistivity >18 MΩ · cm) was used throughout experiments. DNA oligonucleotides were supplied by Metabion GmbH (Germany). Lyophilized pellets were resuspended in 10 mM Tris-HCl buffer, pH 7.8 or DI water (in case of amino-terminated sequence) and stored at−20°C. The corresponding sequences were listed in Table 1.

**Table 1:** Probes, target, and label DNA sequences (5′-3′).

**Functionality**	**Name**	**DNA sequence**
DNA probes	*Probe*	5′-ACTTGCTCGTCTAGATCTGCTCGTTCA-3′
	*Probe-SH*	HS-C6-ACTTGCTCGTCTAGATCTGCTCGTTCA-3′
	*Probe -NH_2_*	H_2_N-C6-ACTTGCTCGTCTAGATCTGCTCGTTCA-3′
Target DNA	*Rand*	5′-AACCATGTTTTGGAAGCCAAGAGCCTA-3′
	*Comp*	5′-TGAACGAGCAGATCTAGACGAGCAAGT-3′
AuNP labels	*Comp-SH*	HS-C6-TGAACGAGCAGATCTAGACGAGCAAGT-3′
	*Rand-SH*	HS-C6-AACCATGTTTTGGAAGCCAAGAGCCTA-3′

*The “C6” abbreviation refers to ω-modified n-hexyl chain attached to terminal 5′ or 3′ oligonucleotide*.

### Instrumentation

Two types of gold transducers have been used in the framework of this studies. Integrated SPRi-Biochips™ made of a high refractive index glass prism coated with gold thin film were purchased in Horiba Scientific (France). Gold disk electrodes were purchased in CH Instruments (USA). SPRi experiments were conducted with the use of SPRi-Lab^+^ instrument (Horiba). Comparative studies of DNA-DNA interactions were performed with the use of homemade microfluidic system, equipped with two parallel cells integrated with optical system. Chronocoulometric and voltammetric measurements were carried out with CHI 1040A potentiostat equipped with homemade voltammetric cell adapted to measurements in nitrogen atmosphere. Apart from gold working electrode, Ag/AgCl/3.0 M KCl reference electrode and gold wire as auxiliary electrode were applied in a classical three-electrode system. Details of instrumentation used for AuNPs characterization (transmission electron microscopy and UV-Vis spectrophotometry) can be found in Supplementary Material.

### Preparation of gold surfaces

Prior to use, SPRi biochips were treated with “alkaline piranha” (NH_3_/H_2_O_2_/water = 1:1:3, v/v) in 60°C for 15 min. After rinsing with DI water, 400 μL of “acidic piranha” (H_2_SO_4_/H_2_O_2_ = 3:1 v/v) was dropped on gold surface. The incubation step lasted for 2 min and then transducers were sequentially rinsed with water, ethanol, and dried in a stream of nitrogen. Gold electrodes of 2 mm diameters were polished with alumina slurry of grain size 0.3 and 0.05 nm, respectively. After mechanical polishing, electrodes were rinsed and sonicated in DI water for 2 min to remove particulate matter. Next, gold electrode surfaces were chemically cleaned by dropping of “acidic piranha” solution prepared as above. After 5 min, electrodes were thoroughly rinsed with DI water. The electrochemical treatment was performed by registration of CV scan series for each electrode in 100 mM solution of sulfuric acid between −0.3 and +1.6 V, until repeatable voltammograms were observed.

### DNA immobilization on gold

In the framework of comparative SPRi and electrochemical studies, three DNA attachment strategies: physical adsorption (DNA-Ads), thiol chemisorption (DNA-SH), and DTC chemisorption were examined. The immobilization of single-stranded deoxyribonucleotide probes (ssDNA) on electrochemical and SPRi transducers was carried out using the same methodology. The derivatization of amine-modified ssDNA was obtained according to procedure described by Sharma (Sharma et al., 2008) with slight modifications (Drozd et al., 2016). In brief, 20 μM solution of *Probe-NH*_2_ in 200 mM phosphate-borate (PB) buffer pH 10.0 was mixed with aliquot of 20 μM (equimolar) solution of CS_2_ in the same buffer and then sonicated in ice bath for 10 min. Then solution of DTC-modified probe was diluted to desired concentration (5 μM) with PB buffer. In case of thiolated (*Probe-SH*) and unmodified (*Probe*) ssDNA, respective solutions in concentrations of 5 μM were prepared directly in 200 mM PB buffer. The immobilization was accomplished by dripping of 25 μL of each solution on freshly cleaned gold electrodes or SPRi transducers. The topography of SPRi biochip typically consists of three separate, vertical strips. Such arrangement was aimed at obtaining of orthogonal pattern after installation of biochip in microfluidic system. The immobilization was carried out for 18 h in humid conditions and at room temperature. Then both types of Au transducers were carefully rinsed with DI water and dried under argon stream.

### Synthesis of citrate-stabilized gold nanoparticles and their modification with ssDNA

Spherical, citrate-capped AuNPs with mean diameter of 13.0 nm were synthesized according to Turkevich method (Turkevich et al., 1951). Modification of AuNPs with thiolated DNA was accomplished by the “salt-aging” procedure described previously by Mirkin et al. (1996). More detailed description of synthetic procedures can be found in Supplementary Material.

### Chronocoulometric determination of DNA surface density

Electrochemical surface areas of gold electrodes were determined by means of cyclic voltammetry according to procedures described by Trasatti and Petrii (1992). A total surface area was given from the charge of gold oxide reduction peak, assuming 390 μC·cm^−2^ as an unit value (Tichoniuk et al., 2010). DNA density on gold were determined based on methodology by Steel et al. (1998). Electrochemical measurements were realized in six repetitions to obtain statistically meaningful results. The charge related to the amount of adsorbed redox marker was calculated according to linearized form of the Anson equation, indicating the proportionality of the charge diffusion component to the square root of time. Experimental details of voltammetric and chronocoulometric measurements and methodology of DNA coverage calculation were extensively described in Supplemental Data.

### SPRi studies of DNA-DNA interactions

The real-time monitoring of DNA-DNA or DNA-ssDNA-functionalized AuNPs (AuNPs@ssDNA) interactions were performed in PBS buffer pH 7.4 containing 0.25 M NaCl as running buffer (RB) under a constant flowrate of 25 μL · min^−1^ for DNA-DNA and 10 μL · min^−1^ for DNA-AuNPs@ssDNA studies, respectively. All measurements in differential mode were realized by simultaneous injection of the complementary target (*Comp*) and random sequence (*Rand*) of equal concentration (within 1 and 1,000 nM range) to separate microfluidic cells. If not stated otherwise, injections typically lasted for 7 min. Before the measurements, three separate fields (spots) for which the reflectance changes were monitored, were defined (separately for each type of receptor layer both flowcells). As regenerating agent (inducing denaturation of DNA duplexes) 50 mM aqueous NaOH was used. To obtain mixed type monolayers (MCH-blocked), repeated injection of 1 mM of MCH in RB for 10 min was carried out. All experiments were conducted in triplicate. Differential sensograms (used throughout quantitative analysis) were obtained by subtracting the normalized values of reflectance changes (ΔR) for the corresponding detection areas in both cells (ΔR_*Comp*_–ΔR_*Rand*_). Values of SPRi responses used for preparation of calibration curves were typically acquired as reflectivity differences between ΔR levels 1 min before and 10 min after beginning of target DNA injection.

## Results and discussion

Reports concerning the utilization of DNA-DTC assemblies as receptor layers usually attribute the attractiveness of analytical parameters of hybridization-based biosensors to stable, bidentate anchoring of DNA probes to gold (Sharma et al., 2008; Wang et al., 2012; Lou et al., 2015). It should be however underlined, that only indirect methods of signal generation were applied in the case of discussed solutions. Therefore, it is difficult to unequivocally assess the significance of probe density resulting from the applied immobilization strategy for a biosensor sensitivity. It should be however underlined, that the proposed approach provides a critical examination of selected methods of oligonucleotide immobilization for construction of DNA biosensing platforms. The major advantage of the SPRi technique used in this work lies in the possibility of direct measurement of the associated mass corresponding to formation of DNA duplexes in the label-free format.

### Comparative characterization of DNA immobilization strategies

**Figure 1:**
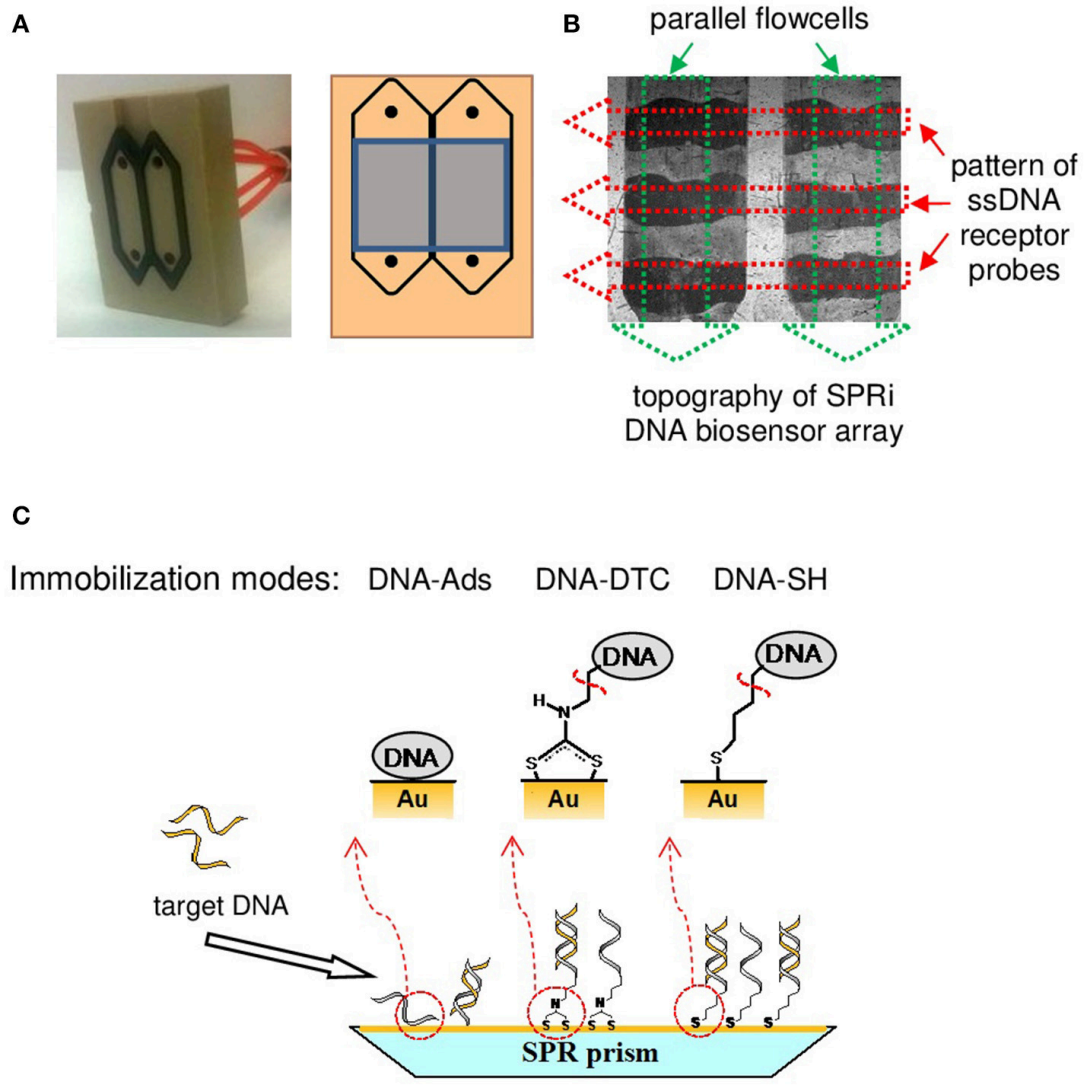
Schematic illustration of SPRi instrumentation and methodology utilized in this work. **(A)** Image of two-channel, homemade SPRi microfluidic cell (left). Scheme of cell design with indicated detection area of SPRi optical system (right). **(B)** Flow cell image captured by CCD camera with the schematic of orthogonal pattern of bioreceptor probes (red arrows) and microfluidic cells (green arrows). **(C)** Schematic representation of the DNA-immobilization strategies examined in the framework of presented studies (magnified side-view of SPRi biochip™).

To comparatively examine SPRi responses corresponding to injections of a model ssDNA sequence, sensograms were registered simultaneously for three types of oligonucleotide receptor layers: thiolate (DNA-SH), dithiocarbamate (DNA-DTC), and physisorbed (DNA-Ads). To assure reliable negative control, corresponding *Rand* sequence was simultaneously injected into reference flowcell. Such approach allows for the effective suppression of the influence of non-specific interactions of target DNA with Au on obtained real-time SPRi sensograms. The design of differential microfluidic cell used throughout our SPR studies is depicted in Figure 1A.

According to literature reports, conversion of oligonucleotides modified with terminal aminolink into DTCs was accomplished following to the spontaneous reaction with CS_2_ in a basic environment (Sharma et al., 2008). According to our previous report, slight modifications of known methodology (reaction at pH 10.0 instead of 9.0 and ultrasonic propagation) were introduced to ensure both the improvement of reaction rate and the efficiency of amine conversion (Drozd et al., 2016). The apparent pattern of immobilized ssDNA probes (dark stripes—see Figure 1B), may act as a proof of the successful immobilization of oligonucleotide by means of chemisorption of both thiolate and DTC as well as a direct physisorption of DNA strands on a gold chip. The details of DNA-tethering chemistry are depicted in Figure 1C.

**Figure 2:**
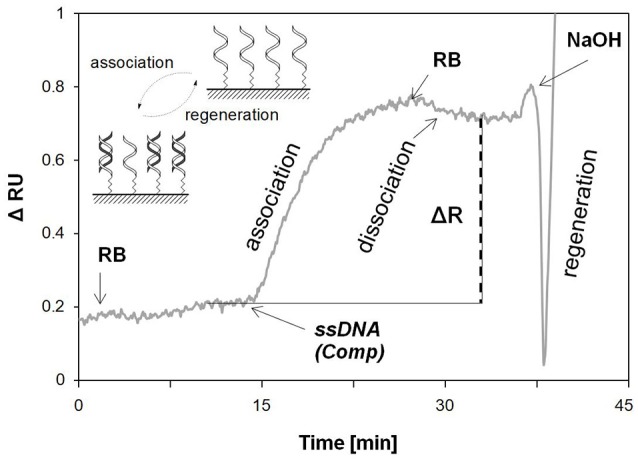
Exemplary, real-time results of DNA-DTC probe hybridization with complementary sequence (*Comp*, C_DNA_ = 50 nM). Differential sensogram was registered for single family of SPRi spots. Particular stages of the SPR experiment and the way of quantitative interpretation of the sensor's response are indicated.

The increase of differential SPRi signal was observed in the response to complementary target sequence (*Comp*) used in the concentration of 50 nM, regardless of immobilization strategy. Result of individual experiment indicating the capability of DNA-DTC for hybridization with complementary sequence, together with the specified steps of SPRi experiment is depicted in Figure 2. First, the reusability of obtained receptor layers was verified by fourfold repetition of target DNA injection (50 nM), each time followed by removal of hybridized strand using 50 mM of NaOH solution in the regeneration step. It is noteworthy, that in any studied case the statistically relevant drop of SPRi response along with subsequent regeneration cycles was not observed. Such result indicates the relevance of non-specific interactions between oligonucleotides and gold surface. The surprising robustness of this construct can be assigned to increased significance of multidentate, adsorptive interactions of amine moieties of nucleobases observed for DNA probes of moderate length. The mechanisms of length- and base-dependent ssDNA adsorption on gold were thoroughly investigated (Steel et al., 2000; Kimura-Suda et al., 2003). Such approach was among others successfully utilized for the development of DNA biosensors and multiplex microplatforms (Koo et al., 2015; Sina et al., 2017). Interestingly, beyond two methods based on chemisorption, also DNA adsorption approach turned out to be sufficient to assure the stable attachment of DNA receptors for further studies in a microfluidic system, where oligonucleotides are continuously exposed to shear forces.

### Analytical performance of various receptor layers toward DNA sensing

**Figure 3:**
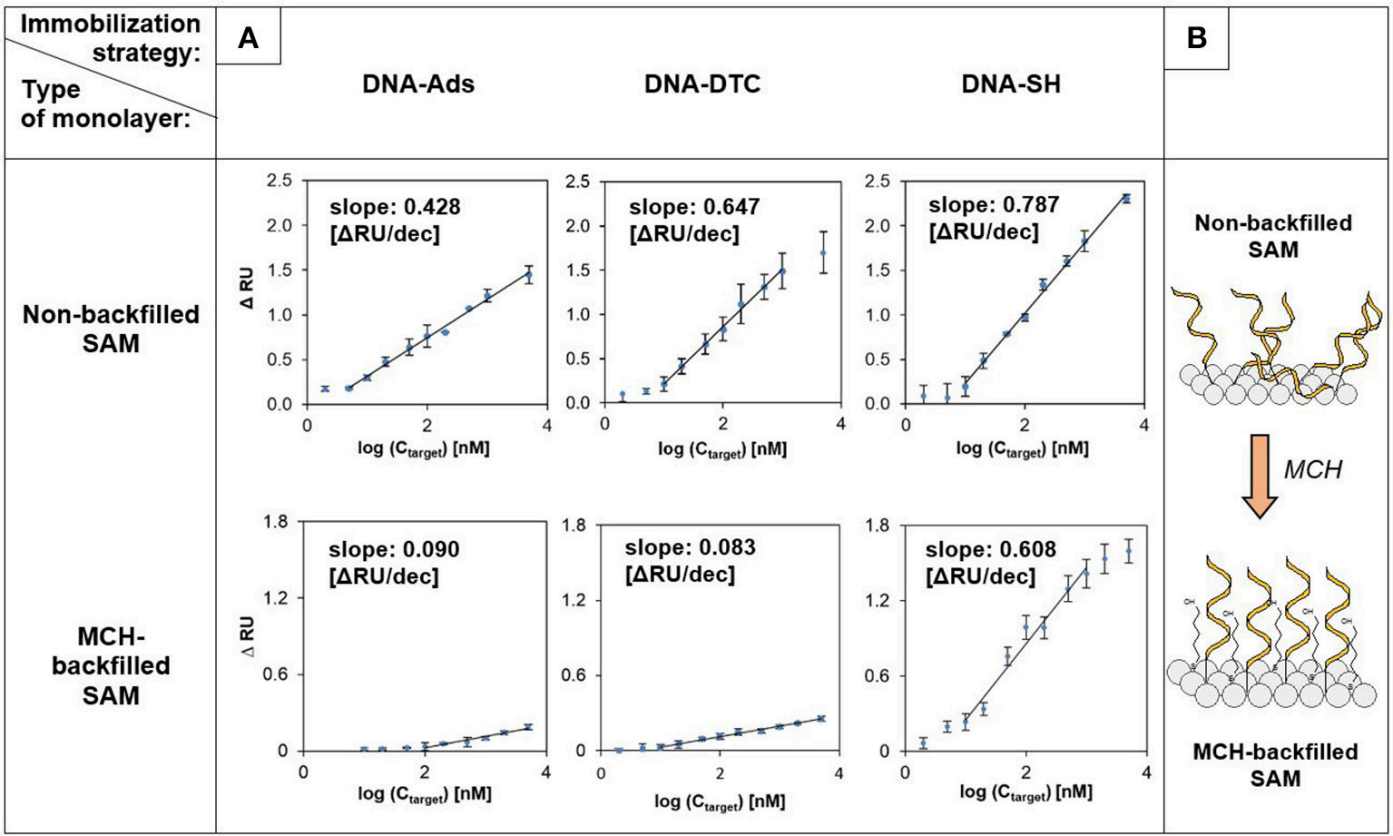
SPRi calibration curves and analytical parameters obtained for various immobilization strategies (*n* = 3)**. (A)** Comparative results for non-backfilled (top row) and MCH-backfilled (bottom row) DNA-SAMs. **(B)** Schematic illustration of DNA-monolayer reorganization induced by MCH. Random sequence (*Rand*) was injected into the reference cell throughout experiments. “*n*” value corresponds to the number of detection areas for which reflectance changes (independent sensograms) were recorded.

Due to the satisfactory reproducibility of the obtained SPRi biosensors, a full range calibration curves based on multiple injections of DNA were prepared (see Figure 3A). Beyond the homogeneous DNA assemblies described above, our research also focused on the determination of analytical parameters of mixed-type receptor layers obtained by backfilling the surface with 6-mercaptohexanol. Introduction of MCH was dictated by its widespread use in electrochemical and SPR biosensors exploiting DNA SAMs (He et al., 2000; Tichoniuk et al., 2010). Main goals of such treatment aimed at obtaining of highly ordered assemblies, backfilling of ssDNA SAM defects and pinholes, and counteracting the non-specific adsorption (Keighley et al., 2008). Schematic representation of influence of MCH on morphology of DNA-based SAM is depicted in Figure 3B.

Obtained relationships between SPR response and logarithm of the target DNA concentration were characterized by the linear course, typical for DNA sensors used in fluidic systems (Hao et al., 2017). In the case of homogeneous layers (without MCH) the highest sensitivity (defined as a slope of the linear section of calibration curve) was noted for the DNA-SH monolayer and amounted to 0.787 of resonant unit per decade. The slope of the curve for DNA-DTC was slightly smaller and was equal to 82.2% of the value obtained for DNA-SH. Surprisingly high sensitivity, which amounted to 54.4% of the value for DNA-SH monolayer was observed for DNA-Ads, taking into account the mechanism of DNA duplex formation, which forces the repeatable conformational changes of the receptor layer. In contrary to several literature reports, this result indicates the existence of a sufficiently strong interaction between oligonucleotide probe (27 nucleobases length) and bare gold, while maintaining good availability of immobilized probes to hybridization with target DNA (Georgiadis et al., 2000; Nelson et al., 2001). It was unambiguously confirmed in a number of literature reports, that intensity of hybridization signals measured in direct format is predominantly determined by the surface density of oligonucleotide receptors (Steel et al., 1998; Peterson et al., 2001). The determined LOD values for examined biosensors amounted to 824, 725, and 615 pM, for DNA-Ads, DNA-SH, and DNA-DTC, respectively. Detection limits were calculated using three standard deviations of the reference signal (Hao et al., 2017). Despite differences in the sensitivities of the homogeneous layers, they are all characterized by wide range of linear response (see Figure 3).

It can be seen in Figure 2, that under applied conditions the time of interaction required to achieve the state of dynamic equilibrium is longer than the time applied for injection of target DNA. The relatively short contact time of DNA probes with target sequence (7 min) is insufficient to achieve the equilibrium of the DNA-DNA duplex formation at association step. It should be stressed, that this fact opens up some possibilities for optimization of injection time in order to further improve the analytical parameters of obtained biosensors.

At the next stage of studies, the impact of MCH-backfilling on the working parameters of various DNA-based receptor layers was revealed. DNA-SH monolayer turned out to be the most resistant to influence of 6-mercaptohexanol, which induced the loss of sensitivity by 22.7%. The observed sensitivity drop can be assigned to the partial desorption of probe DNA, caused by chemisorption of competitive surface-active agent (MCH) (Steel et al., 2000). The main difference compared to homogeneous layers was the significant decrease in the sensitivity of DNA-Ads (drop of calibration curve slope to 21.0% of the initial value) and DNA-DTC (sensitivity reached only 12.8% of the initial value obtained for the corresponding homogeneous layer). The latter result should be considered disappointing, as it indicates that the durability of the DTC-Au assembly to thiol-induced desorption turned out to be significantly lower than expected.

The introduction of backfilling agent was also reflected in the calculated detection limits. Values of LOD for developed biosensors amounted to 2.88 nM for DNA-Ads, 662 pM for DNA-SH, and 1.96 nM in the case of DNA-DTC. In the case of DNA-SH, modification with MCH induced the rearrangement and ordering of the monolayer, with a small contribution of desorption of bioreceptor. This resulted in the slight improvement of detection limit (725–662 pM). Conversely, in the case of DNA-Ads and DNA-DTC the effect of receptor desorption presumably turned out to be dominant, which was reflected in about 3.5-fold (DNA-Ads) and 3.2-fold (DNA-DTC) increase of LOD value.

Basic conclusions arising from presented research remain in opposition to few available literature reports. Comparative stability studies, which were described for DTC- and thiolate-based SAMs unequivocally indicate the higher energy of DTCs chemisorption on gold in comparison to thiol assembly (Park et al., 2010; Raigoza et al., 2012). It should be however underlined, that their scope of work was limited only to simple, N,N-disubstituted DTC derivatives. Such compounds differ significantly from DTC-DNA utilized in this work in both size of side chain and the order of amine precursor. What is also important, simple, N,N-disubstituted DTCs are routinely purified before being used to form monolayers, which is not possible in the case of N-monosubstituted DTCs. Inability to isolate DNA-DTC from the reaction mixture may significantly impede the formation of monolayers on gold. The adverse effect of co-adsorption of CS_2_ and parent amines on formation of DTC layers was already reported for simple, bifunctional DTCs (Zhao et al., 2005; Adak et al., 2010).

Process of DTC chemisorption may also be disturbed by the interaction of oligonucleotide chain with the gold surface. Assumptions drawn for DNA-DTCs are additionally supported by reports describing the mechanism of formation of thiolated DNA monolayers. The occurrence of non-specific DNA adsorption in coexistence with DNA-SH assembly was already described by Steel et al. (2000). According to this work, for thiolate monolayers composed of long oligonucleotide chains (over 24 nb), when the length of the immobilized DNA chain increased, the degree of order of obtained SAMs gradually decreased. Such phenomenon was explained by the increasing contribution of non-covalent interactions of nucleobases with Au. It should be also pointed out, that Morf et al. (2006) indicated an approximately 4-fold slower kinetics of DTC assembly compared to thiol monolayers. Low rate of Au-DTC bond formation may further enhance the interferences caused by co-adsorption of DNA chains.

### Chronocoulometric determination of surface density of immobilized DNA probes

As postulated in section Analytical Performance of Various Receptor Layers Toward DNA Sensing, the putative explanation of diversity of biosensors sensitivity lies in the substantial differences in density of DNA immobilized on gold. For this reason, such assumption was verified by determining the surface coverage of oligonucleotide probes immobilized on Au. Chronocoulometric method of DNA quantification based on the measurement of the charge corresponding to reduction of Ru(NH_3_${)}_{6}^{3+}$ adsorbed by polyanionic DNA chains bound to surface of gold electrodes was applied (Steel et al., 1998; Tichoniuk et al., 2010) (see Figure S1). To assure reliability of chronocoulometric measurements, DNA monolayer without defects and pinholes is required, therefore the scope of research was limited to MCH-backfilled, mixed monolayers.

As predicted, DNA-SH/MCH monolayer was characterized with the highest amount of immobilized DNA molecules. The obtained probe density of 7.62 · 10^12^ cm^−2^ stays in accordance with values known from the literature. According to them, thiolated SAMs prepared in similar way exhibit surface density within the range of 1-10·10^12^ cm^−2^ (Peterson et al., 2001; Tichoniuk et al., 2010). Values within these limits are widely recognized as optimal in terms of density of oligonucleotide-based SAMs dedicated to the detection of complementary DNA sequences. On the other hand, the remaining bioreceptor layers were characterized by several times smaller surface coverage, as in the case of mixed DNA-DTC which was characterized with the lowest density (1.47 · 10^12^ molecules · cm^−2^), over 5 times lower than in the case of corresponding DNA-SH (see Table 2). It is noteworthy, that similar trend was observed for alkyl DTC and thiolate monolayers (ferrocene-C16-DTC and ferrocene-C16-SH) by Eckermann et al. (2010), where the ratio of the surface densities calculated for thiolate and DTC monolayers was 3.46. Lower surface density of DTC derivatives is typically explained by the occurrence of steric hindrance resulting from bidentate Au-DTC chelation and thus attenuation of hydrophobic interactions of alkyl chains (Eckermann et al., 2010; Raigoza et al., 2012). It should be however noted, that surprisingly high density of DNA-Ads, together with relatively high stability of adsorbed DNA additionally confirms the assumption of large contribution of co-adsorption in the overall effect observed for DTC monolayers. Such conclusions remain in substantial discrepancy to reports concerning DNA-DTC monolayers in construction of biosensing platforms, in which the advantages of bidentate chemisorption of DTC are indicated *a priori*, without excessive verification of their superiority over thiolate chemisorption and physisorption.

**Table 2:** Surface densities of DNA oligonucleotides immobilized on the surface of gold disk electrodes (*n* = 6).

**Monolayer composition**	**DNA-Ads /MCH**	**DNA-SH/MCH**	**DNA-Ads/MCH**
DNA surface density (cm^−2^)	(1.63 ± 0.30) ·10^12^	(7.62 ± 0.59) ·10^12^	(1.47 ± 0.40) ·10^12^

*“n” value corresponds to the number of electrodes used*.

### Correlation between DNA surface coverage and efficiency of AuNPs labeling

The obtained analytical parameters of DNA-DTC based SPRi biosensors in the label-free format do not surpass the corresponding, thiol-based ones, which is attributed to lower surface density of receptors. However, the basic difference between the research presented in this work and the mentioned literature reports lies in the fact, that described DTC-DNA-based biosensors exploited indirect approach for analytical signal generation (Wang et al., 2012; Lou et al., 2015). This discrepancy became the motivation to investigate the correlation between the surface density of oligonucleotide receptors and labeling efficiency using DNA-functionalized gold nanoparticles. For this purpose, AuNPs functionalized with *Comp* (AuNPs@*Comp-SH*) and *Rand* sequence were utilized as plasmonic signal enhancers. Analysis of TEM micrographs (Figure S2) and position of LSPR maximum (519 nm, see Figure S3) confirmed, that the average diameter of the AuNPs is 13.0 nm (Haiss et al., 2007).

**Figure 4:**
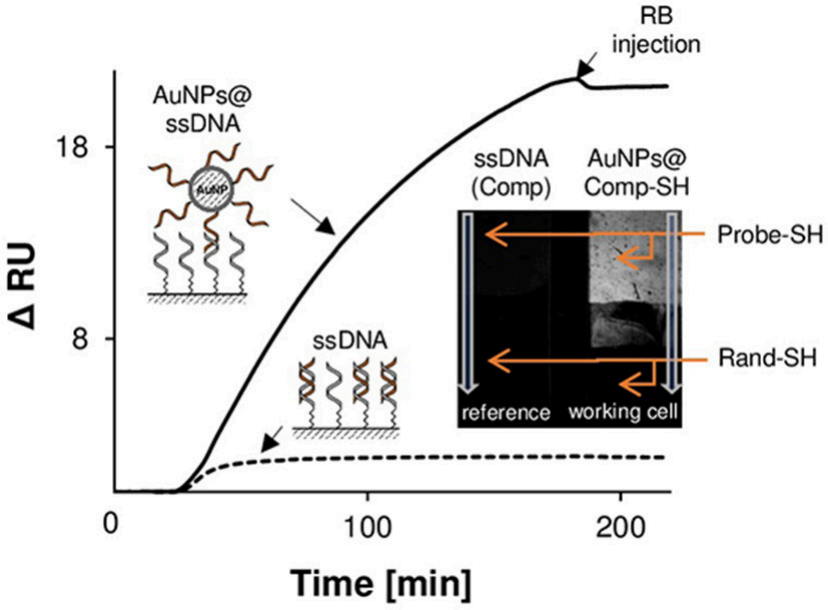
Effect of SPRi signal amplification by means of AuNPs@ssDNA labels. SPR response showing the kinetics of interaction between the exemplary DNA receptor layer (DNA-SH) and the complementary sequence (dashed line) and AuNPs functionalized with complementary sequence (solid line). Insert outlines the differential image captured by CCD camera, showing the surface topography of the SPR sensor and illustrating the performance of AuNPs-induced SPR signal enhancement.

It could be expected that the major difference in dimensions of AuNPs@*Comp-SH* and free *Comp* sequence would be reflected in the kinetics of their binding by capture DNA. To verify such hypothesis, investigations with the use of mixed thiol monolayers (DNA-SH/MCH) were carried out. The experiment consisted of prolonged (160 min), parallel injection of *Comp* (100 nM), and AuNPs@C*omp-SH* (C_AuNPs_ = 320 pM). Schematic diagram of dsDNA duplexes formation in both modes, obtained SPR sensograms and CCD image of SPRi sensor after association step are outlined in Figure 4. Analysis of real-time sensograms indicates the obvious, about 11.5-folds higher SPRi response caused by attachment of AuNPs labels instead of unmodified ssDNA. Observed enhancement effect derives from a double role of AuNPs, which strongly interact with the surface plasmons of gold transducer both by specific plasmonic coupling, as well as association of a relatively large mass of the nanoparticle (He et al., 2000). Significant differences in kinetics of examined interactions are indicated by character of SPRi responses. ssDNA injection resulted in reaching DNA association/dissociation equilibrium after 40 min, whereas in case of AuNPs@*Comp-SH*, such effect was not obtained within the time of experiment (See Figure 4).

As can be concluded from inset of Figure 4, binding of AuNPs labels to DNA-SH monolayer proceeds only through specific DNA-DNA interactions. Lack of observed reflectance shift for random sequence (bottom of working cell) confirms the negligible contribution of non-specific interactions between AuNPs and non-complementary DNA. A suppression of non-specific adsorption is promoted by electrostatic repulsion between polyanionic DNA chains of receptor layers and functionalized AuNPs. Similar effect was observed for mixed DNA-Ads and DNA-DTC monolayers. This observation opened the possibility of their application in indirect DNA detection using AuNPs as plasmonic labels.

**Figure 5:**
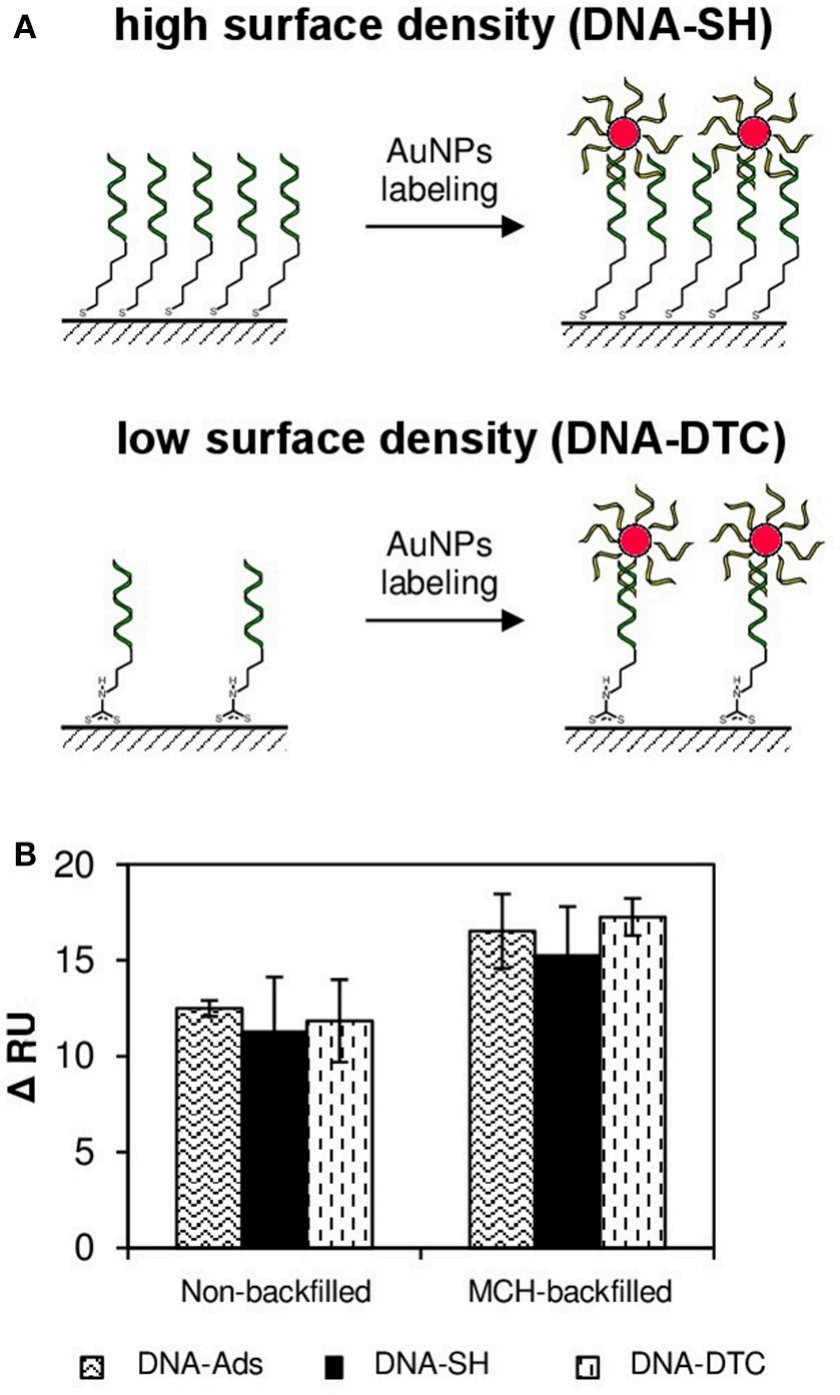
The influence of DNA probe surface density on efficiency of AuNPs-induced SPR signal enhancement **(A)** Schematic illustration of the mechanism of DNA-AuNPs@DNA duplexes formation on the example of monolayer of high DNA density (DNA-SH) and low DNA density (DNA-DTC). **(B)** The SPR responses of various DNA receptor layers toward injection of AuNPs@*Comp-SH* labels. AuNPs modified with thiolated random sequence (*Rand-SH*) were injected into the reference cell throughout experiments.

The occurrence of steric hindrances and hampered transport of mass result in a slower kinetic of gold nanoparticle conjugates association and thus lowering of labeling efficiency. Therefore, it can be concluded, that the use of nanomaterial-based conjugates with the large spatial dimensions should imply the independence of the values of SPR signals from the surface coverage of DNA probes. This effect was schematically illustrated by the example of DNA-SH and DNA-DTC monolayers in Figure 5A.

As the next step, the thoughtful examination of relationship between spatial dimensions of AuNP labels and surface density of DNA receptor layers was carried out. Literature reports point to the stiffness of DNA double helix sufficient enough to form rigid “bridges” between solid substrate and gold nanoparticle of diameter up to 200 nm (Jones et al., 2015). Therefore, it can be expected that AuNPs used throughout the experiment form stable duplexes with immobilized DNA probes as schematically depicted in Figure 5A. Based on this assumption, ratios of the axial section of single Au nanoparticle and the area per immobilized ssDNA receptor were determined. The calculated area of the projection of average nanoparticle on plane (axial section of 13.0 nm diameter, assuming its sphericity) equals to 132.7 nm^2^. In turn, the average surface area per single DNA molecule in DNA-SH/MCH monolayer is 13.12 nm^2^ (based on values of surface density listed in Table 2 and assuming the uniformity of surface coverage). Obtained results (ratio = 10.11) indicate, that at least 10 *Probe-SH* molecules belong to single nanoparticle label. It is easy to notice, that for each of the mixed-type monolayers examined in this work, the number of DNA probe molecules per single label molecule is greater than one (10.11 for DNA-SH, 2.16 for DNA-Ads, and 1.95 for DNA-DTC, respectively). Obviously, such ratios are significantly underestimated, because in the calculated values complete coverage of surface with spherical nanoparticles was assumed and the contribution of oligonucleotide ligands (the increase in AuNPs hydrodynamic radius and electrostatic interactions) were not taken into account. Nevertheless, it can be stated, that in each studied case the packing density of DNA probes makes impossible to bind the theoretical number of AuNPs labels due to steric limitations. Therefore, surface density of mixed DNA-Ads, DNA-DTC, and DNA-SH layers should not significantly affect the amount of bound AuNPs labels and thus the SPRi response.

The efficiency of AuNPs@*Comp-SH* binding to all types of obtained monolayers was experimentally verified. Values of reflectance shifts (ΔRU) obtained on the basis of differential sensograms are presented in Figure 5B. As expected, response values resulting from hybridization of AuNPs@*Comp-SH* with oligonucleotide probes proved to be similar, regardless of DNA immobilization strategy. It should be underlined, that no decrease of sensitivity of mixed DNA-DTC and DNA-Ads monolayers (which was typical phenomenon in the case of label-free formats) was not observed. Presented results point to the conclusion, that DNA monolayers of low density can compete with dense thiol monolayers in terms of sensitivity only in the case biosensors exploiting strategy of indirect signal generation, as documented in few reports regarding DNA biosensors (Sharma et al., 2008; Lou et al., 2015).

### Efficiency of SPRi response amplification in the competitive format

In the final step, proof of principle of AuNPs application in DNA detection system based on competitive format was presented. The mechanism of indirect detection in the competitive system exploits the decrease of surface density of receptors available for labeling, due to primary interaction with the analyte. The goal of this study was the verification, whether the use of receptor layer of the low density (more compatible to label dimensions, as proven above) may contribute to the improvement of labeling efficiency of receptors, which are not involved in the interaction with analytes.

**Figure 6:**
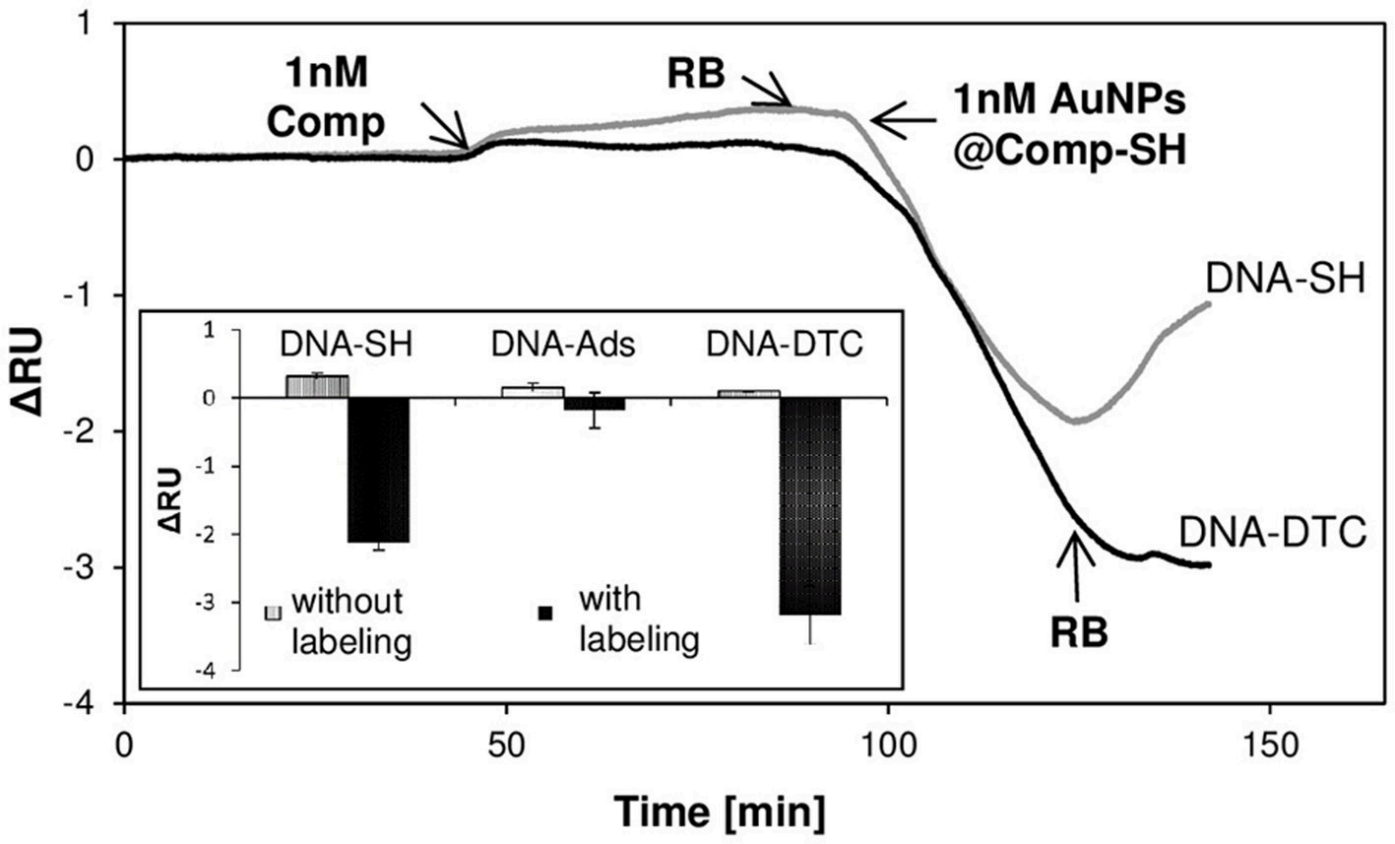
The performance of SPR response enhancement by introduction of AuNPs labels in competitive assay. Exemplary SPRi sensograms illustrating the real-time responses after subsequent injection of *Comp* sequence and AuNPs@*Comp-SH* label. Inset shows the values of total RU shifts calculated before (gray bars) and after labeling in competitive mode (black bars). *Rand* sequence (1 nM) and AuNPs@*Comp-SH* label (1 nM) were simultaneously injected into the reference cell, respectively.

Figure 6 shows exemplary SPRi response to injection of solutions of *Comp* (1 nM), followed by competitive labeling step by means of AuNPs@*Comp-SH* for DNA-SH/MCH and DNA-DTC/MCH monolayers. As expected, in the case of such format, negative responses were obtained, which is caused by the fact, that ssDNA-functionalized AuNPs compete for receptors with ssDNA used as the model target. Inset shows values of corresponding responses obtained before and after signal amplification for all examined biosensors. As expected, the injection of AuNPs@*Comp-SH* induced significant decreases in SPRi responses for DNA-SH/MCH and DNA-DTC/MCH based biosensors. The ratio of signals before and after labeling was 6.6 for thiolated-SAM and 32.0 for DTC-SAM, thus confirming the predicted performance of receptor layer of low surface coverage in efficient binding of AuNPs labels. It is noteworthy, that there was no noticeable enhancement effect for DNA-Ads/MCH, despite its proven ability to bind AuNP labels in the direct format (section Correlation between DNA Surface Coverage and Efficiency of AuNPs Labeling). The possible reason for such behavior of physisorbed DNA is the orientation and surroundings of free ssDNA strands. In contrary to covalently immobilized probes, adsorbed oligonucleotides tend to form more compact, and hence less exposed receptor layer, due to multiple binding sites with bare Au. Partial hybridization with *Comp* renders the remaining ssDNA probes less accessible to bulky AuNP@*Comp-SH* conjugate (Steel et al., 2000). The putative mechanism covers both the enhancement of electrostatic repulsion of polyanionic label conjugates by dsDNA, as well as emergence of a steric hindrance. Formation of helices enforces the rearrangement of monolayer and thus the override of free ssDNA probes by rigid dsDNA duplexes.

LSPR field as the evanescent wave is relatively strong in a distance not exceeding 10 nm and decays up to 30 nm (Hao et al., 2017). Therefore, AuNPs labels retain the unique capability of SPR signal enhancement by electronic coupling only at specified, small distance from Au transducer (He et al., 2004; Sendroiu et al., 2011). It should be considered, that the calculated length of double helix structure composed of 27 base pairs (length of complimentary segment in presented studies) is 7.26 nm, so it seems reasonable from the point of view of sensitivity to eliminate any redundant spacers increasing distance between transducer and plasmonic label (Ho, 1994). In this context, SPR detection in competitive mode seems to be particularly attractive. In such approach distance between AuNPs and Au transducer is usually smaller than in classical sandwich format.

## Conclusions

Three methods of immobilization of DNA oligonucleotides on gold, which find application for SPRi-based DNA biosensing in both label-free and nanoparticle-enhanced format, were critically compared. Studies focused on the evaluation of biosensors with DNA-receptor layers obtained by chemisorption (by means of DTC- and thiolate-assemblies) and physisorption. Obtained DNA receptor layers demonstrated ability to reversibly bind the model complementary sequence, thus providing the wide dynamic range of SPRi response. DNA-SH assemblies turned out to be the most attractive in terms of sensitivity, in the case of both homogeneous and mixed monolayer. The surprising susceptibility of DNA-DTC for desorption under the influence of the surface-active MCH was explained in terms of relatively high contribution of non-specific interactions of DNA chain in during the monolayer formation. In the case of label-free DNA detection based on double helix formation, the surface coverage of immobilized DNA probes was confirmed as the crucial factor influencing the sensitivity of biosensors. Studies on interactions of receptor layers with ssDNA functionalized AuNPs contribute to the explanation of relationship between surface density of DNA receptors and the efficiency of labeling. It was shown, that the surprisingly high efficiency of AuNPs binding even by the monolayers of the low density (DNA-DTC/MCH and DNA-Ads/MCH) can be attributed to relatively low kinetics and large spatial dimensions of AuNPs in relation to the distance of oligonucleotide receptors on gold surface. In the final section, possibility of DNA-DTC/MCH application in AuNPs-amplified SPRi detection of model complementary sequence was demonstrated. We believe, that discussed immobilization approaches should facilitate the development of biosensors for simultaneous detection of multiple specific DNA targets. Due to capabilities offered by SPR measurements in imaging mode, proposed solutions also provide great potential for applications in SPRi-based DNA microarrays.

## Author contributions

MD conducted all the experiments and wrote the draft of the paper; MP provided general guidance, helped in the redaction, and revision of the final version of manuscript; EM supervised the project and reviewed the manuscript.

### Conflict of interest statement

The authors declare that the research was conducted in the absence of any commercial or financial relationships that could be construed as a potential conflict of interest.
