# Supplementary material for: SPRi-Based Biosensing Platforms for Detection of Specific DNA Sequences Using Thiolate and Dithiocarbamate Assemblies
Source: Front Chem. 2018 May 22;6:173. doi: 10.3389/fchem.2018.00173 (PMC5972272; doi:10.3389/fchem.2018.00173)
Supplement: Supplementary file 1 [file Data_Sheet_1.docx]

***Supplementary Material***

**SPRi-based biosensing platforms for detection of specific DNA sequences with a use of thiolate and dithiocarbamate self-assemblies**

Marcin Drozd^a^, Mariusz Pietrzak^a^*, Elżbieta Malinowska^a,b^

a) Warsaw University of Technology, Faculty of Chemistry, The Chair of Medical Biotechnology, Noakowskiego 3, 00-664 Warsaw, Poland

b) CEZAMAT PW, Poleczki 19, Warsaw, Poland

*Corresponding author. Tel.: +48 222 347573, Fax: +48 226 282741.

E-mail address: mariusz@ch.pw.edu.pl

**Experimental section:**

*Chemicals*

Gold(III) chloride trihydrate, sodium citrate dihydrate, tris(2-carboxyethyl)phosphine hydrochloride, sodium chloride, potassium chloride, sodium hydrogen phosphate and potassium dihydrogen phosphate were obtained from Sigma-Aldrich (Poland) and used as received. Nitric acid (68.5%), hydrochloric acid (37%) and sodium hydroxide were purchased in Avantor S.A. (Poland). All chemicals used in this work were at least analytical grade.

*Synthesis of citrate-stabilized gold nanoparticles and their modification with ssDNA*

Spherical, citrate-capped AuNPs with mean diameter of 13.0 nm were synthesized according to Turkevich method (Turkevich et al., 1951). Before synthesis all glassware used throughout was treated with *aqua regia* (mixture of nitric acid/hydrochloric acid, 1:3 (v/v)) and washed with DI water. Briefly, to 96 mL of boiling, 1.04 mM aqueous solution of HAuCl_4_ 4 mL of 100 mM solution of sodium citrate was rapidly injected and the whole mixture was further maintained in boiling temperature until characteristic, ruby-red color turned out. Modification of AuNPs with thiolated DNA was accomplished by the procedure described by Mirkin et al., (1996), using 1 mL of previously prepared AuNPs, 1 mL of 3 μM solution of *Comp-SH* or *Rand-SH* DNA sequence in 10 mM phosphate buffer, pH 7.6, containing 100 μM tris(2-carboxyethyl)phosphine (18 hours of injection) and 2 mL of 1 M NaCl as “salt-aging” agent (70 hours of injection). As prepared AuNPs were purified via triplicate centrifugation (25 min x 18 000 rpm) and then resuspended in PBS buffer. The calculated concentrations of ssDNA-capped AuNPs in stock solutions were adjusted to 16.0 nM (according to UV-Vis method by Haiss et al., (2007)).

*Characterization of AuNPs and AuNPs@ssDNA*Absorption spectra of AuNPs were acquired with a Perkin-Elmer Lambda 25 spectrophotometer in range of 200-800 nm. TEM micrographs of gold nanoparticles were captured with the use a JEOL S5500 transmission electron microscope operating at an accelerating voltage of 30 kV. Mean diameter of AuNPs was calculated based on analysis of UV-Vis absorption spectra according to correlation described by Haiss et al., (2007) and analysis of representative TEM micrographs (at least 150 nanoparticles taken into account).

*Chronocoulometric determination of DNA surface density*

Electrochemical surface areas of gold electrodes were determined by means of cyclic voltammetry. Four CV scans between -0.8 V and 1.6 V at scan rate 0,1 V∙s^-1^ in 50 mM phosphate buffer pH 7.6 were registered for each electrode. DNA densities on gold were determined based on methodology previously described by Steel et al., (1998). The basis for determining the electrochemical surface of electrodes was the value of charge associated with the reduction of gold (equivalent to the peak area of repetitive CV voltammograms). In our calculations value 390 µC∙cm^-2^ was used as the charge corresponding to the unit Au surface (Trasatti and Petrii, 1991).

Prior to electrochemical measurements, surfaces of DNA-modified electrodes as well as bare ones were backfilled by incubation in 1 mM solution of MCH in phosphate-borate buffer, pH 10.0 for 10 min. After thoroughful rinsing with DI, electrodes were immersed in supporting electrolyte (10 mM Tris-HCl, pH 7.4, without RuHex) and then reductive potential step from 0.1 to -0.4 V *vs*. Ag/AgCl for 1 s was applied. Next, a similar cycle of incubation and chronocoulometric measurement was repeated in an electrolyte containing redox marker (10 mM Tris-HCl, pH 7.4, 100 mM RuHex). All CC experiments were performed under N_2_ atmosphere and preceded by nitrogen purging of background electrolytes for 10 min. Electrochemical measurements were realized in six repetitions to obtain statistically meaningful results.

The main calculations were performed on the basis of Anson equation (Eq. 1). Intercepts obtained for DNA-modified electrodes (Eq. 1b) correspond to the sum of the capacitive charge of double layer (Q_dl_) and the charge of adsorbed layer (nFAΓ_0_). Subsequently, the Q_dl_ component was suppressed by subtraction of current values ​​registered for electrodes modified with DNA and the same electrodes modified only by MCH solution. The set of chronocoulometric curves obtained for a single electrode was shown in Figure S1. The final step was the chronocoulometric determination of the surface density of adsorbed RuHex (Eq. 2).

y

a

=

b

x

+

•

(1)

•

$Q=\frac{{2nFACoDo}^{1/2}}{\pi^{1/2}}$ $t^{1/2} + Q_{dl}+nFA\Gamma_{0}$

$\boldsymbol{Q}_{\boldsymbol{ads}}\boldsymbol{=}\left( \boldsymbol{Q}_{\boldsymbol{4}}\boldsymbol{-}\boldsymbol{Q}_{\boldsymbol{3}} \right)\boldsymbol{-(}\boldsymbol{Q}_{\boldsymbol{2}}\boldsymbol{-}\boldsymbol{Q}_{\boldsymbol{1}}\boldsymbol{)}$

(2)

where:

Γ – surface coverage,

Q_1_ – charge for MCH-modified electrode, measurement in buffer without RuHex,

Q_2_ – charge for MCH/DNA-modified electrode, measurement in buffer without RuHex,

Q_3_ – charge for MCH-modified electrode, measurement in buffer with RuHex,

Q_4_ – charge for MCH/DNA-modified electrode, measurement in buffer with RuHex,

n — the number of electrons participating in redox reaction: 1 (Ru^3+^ → Ru^2+^),

F − Faraday’s constant: 96485,3 C·mol^-1^,

A – electrochemical surface area,

Surface coverage was determined on the basis of the assumption of proportionality of DNA quantity to the amount of adsorbed redox marker, as described by formulas 3 and 4 (Steel et al., 1998):

(4)

(3)

| $\Gamma_{DNA}=\Gamma_{RuHex}\cdot\frac{z}{m}\cdot N_{A}$ | $\Gamma_{RuHex}=\frac{Q_{ads}}{nFA}$ |
| --- | --- |

$\Gamma_{\mathrm{DNA}}=\Gamma_{\mathrm{RuHex}}\cdot\frac{z}{m}\cdot N_{A}$

where:

z –RuHex charge: 3,

m – number of nucleobases: 27,

N_A_ – Avogadro number.

**Results and discussion:**

**Figure S1.** Exemplary chronocoulometric Anson plots for DNA-DTC/MCH-modified electrode.

$$nFA\Gamma_{0}$$

**Figure S2.** TEM image of AuNPs used as plasmonic labels.


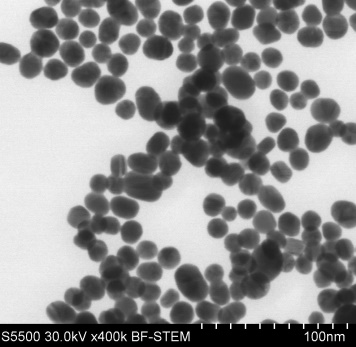


**Figure S3.** UV-Vis spectra of AuNPs before (solid line) and after (dashed line) modification with ssDNA (*Comp-SH*).

519 nm

525 nm

AuNPs@ *Comp-SH*

AuNPs@ citrate

**References**

Mirkin, C.A., Letsinger, R.L., Mucic, R.C., and Storhoff, J.J. (1996). A DNA-based method for rationally assembling nanoparticles into macroscopic materials. Nature, 382, 607-609

Haiss, W., Thanh, N.T., Aveyard, J., and Fernig, D.G. (2007). Determination of size and concentration of gold nanoparticles from UV-vis spectra. Anal. Chem. 79, 4215-4221. doi: 10.1021/ac0702084

Steel, A.B., Herne, T.M., and Tarlov, M.J. (1998). Electrochemical quantitation of DNA immobilized on gold. Anal. Chem. 70, 4670–4677. doi: 10.1021/ac980037q

Trasatti, S., and Petrii, O.A. (1992). Real surface area measurements in electrochemistry. J. Electroanal. Chem. 327, 353-376. doi: 10.1016/0022-0728(92)80162-W

Turkevich, J., Stevenson, P.C., and Hiller, J. (1951). A study of the nucleation and growth processes in the synthesis of colloidal gold. Disc. Faraday Soc. 11, 55-75. doi: 10.1039/DF9511100055
